# Hydrogen Removal from Fe at Room Temperature: A Study on Hydrogen Trapping Mechanisms

**DOI:** 10.3390/ma19091903

**Published:** 2026-05-06

**Authors:** Kun Zhang, Honglei Li, Denggao Guan

**Affiliations:** College of Materials and Chemistry & Chemical Engineering, Chengdu University of Technology, Chengdu 610059, China; zkyl2468@163.com (K.Z.);

**Keywords:** hydrogen, trap sites, electro-oxidation technique, thermal desorption spectroscopy

## Abstract

A proper understanding of hydrogen–trap interactions in materials is of considerable significance, as it holds the potential to provide promising solutions to the long-standing issue of hydrogen embrittlement. In the present study, we employed a novel integrated approach combining electro-oxidation (EO) technique and thermal desorption spectroscopy (TDS) to characterize both reversible and irreversible deuterium in Fe samples. The samples were deuterium-charged at 500 kPa and temperatures ranged from 25 °C to 500 °C. Deuterium retention was measured by TDS for samples with and without EO treatment. Experimental findings demonstrate that the EO technique not only accelerates the expulsion of spontaneously releasable deuterium but also efficiently removes the majority of non-spontaneously releasable deuterium. It is evidenced that the proportion of reversible deuterium in the non-spontaneously releasable deuterium fraction reaches as high as 70%. Furthermore, an illustrative energy level diagram concerning the different barriers depending on the trap sites was devised to elucidate the trapping and diffusion behaviors of deuterium. Correspondingly, the microstructural trap sites associated with reversible or irreversible states were discussed in detail. This work enhances our understanding of hydrogen-Fe material interactions, thereby strengthening the fundamental theories underlying hydrogen embrittlement.

## 1. Introduction

Hydrogen has emerged as a substance of great significance in the modern industrial and energy landscapes. It is widely utilized in a plethora of industrial processes and holds the promise of being a key energy carrier for the future sustainable society [1]. In the field of hydrogen energy, the storage of high-press hydrogen is an important task involving safety [2,3]. Hydrogen embrittlement (HE) is a phenomenon that occurs when excessive hydrogen penetrates into the lattice structure of metals [4,5,6,7]. This can lead to a significant reduction in the ductility and tensile strength of the metal. In the case of high strength steels, which are commonly used in structural applications, HE can be particularly severe [8,9]. For example, in the oil and gas industry, pipelines made of high strength steels are at risk. Once embrittlement occurs, the pipelines become more susceptible to cracking and failure, which can lead to catastrophic leaks and environmental disasters [10,11,12,13]. Although the phenomenon was first identified a century ago, HE remains an extremely important unsolved industrial challenge.

To address HE issues, scientists try to understand the hydrogen–trap interactions in materials and, up to now, have proposed several HE theories [14,15,16,17,18]. These theories still cannot fully explain all HE behaviors. Moreover, the lack of direct experimental characterization regarding such hydrogen–trap interactions makes fundamental understanding of hydrogen-induced damage elusive [19]. Some studies introduce hydrogen trap sites to elevate critical hydrogen concentrations for embrittlement resistance; however, the reported critical concentration values show significant discrepancies, and this strategy does not consistently achieve the desired effect [20]. Some possible reasons include hydrogen mobility due to its reversible traps with low binding energy. Moreover, the ratio of reversible to irreversible hydrogen varies significantly across different materials. It should be noted that reversible hydrogen, in contrast to irreversible hydrogen trapped with high binding energy, is responsible for HE behaviors. These pose a significant challenge to characterizing HE-related hydrogen interactions in materials.

In case of the characterization on hydrogen–trap interactions, a conventional method is thermal desorption spectroscopy (TDS). This method quantifies total hydrogen release and estimates trap energies via Kissinger-derived models but suffers from significant limitations: it cannot attribute desorption peaks to specific microstructural features and oversimplifies de-trapping as a single-step process, ignoring re-trapping dynamics between adjacent sites [21,22]. As another approach reported by Ming Au, Tritium autoradiography, while effective for visualizing hydrogen distribution (e.g., at grain boundaries or carbides), is hindered by radioactivity hazards and detects only immobile hydrogen in strong traps [23]. Recent breakthroughs in nanoscale secondary ion mass spectrometry (NanoSIMS) and atom probe tomography (APT) enable direct atomic-scale observation of hydrogen/deuterium at dislocations, grain boundaries, and precipitates [24,25]. However, these techniques have a small view field and restrict simultaneous analysis of multiple trap types under identical conditions, and its destructive nature precludes tracking hydrogen dynamics (e.g., desorption/re-trapping) in the same microstructure [26]. Crucially, techniques like NanoSIMS, APT, and tritium methods predominantly detect strongly trapped hydrogen, neglecting reversible hydrogen known to be mainly responsible for embrittlement. This leads to the fact that the HE mechanism remains incompletely understood. Therefore, it is highly necessary to develop another technical method for characterizing the interactions between hydrogen and traps.

In this study, we employed an integrated approach combining electro-oxidation (EO) and TDS to characterize both reversible and irreversible hydrogen in metallic materials. Herein, we operationally define “reversible” hydrogen as the component removable by the EO process, and “irreversible” hydrogen as the residual component. The EO at room temperature selectively oxidizes reversible hydrogen atoms at the metal surface under anodic polarization, enabling their quantitative removal into an alkaline electrolyte. The TDS measures total hydrogen content by monitoring desorption profiles during controlled heating. By comparing TDS results of two identical samples (one with EO treatment, the other without), one can obtain information about both types of hydrogen. This synergistic methodology provides a comprehensive framework for dissecting complex hydrogen trapping mechanisms in metallic materials.

During the experiment, we charged Fe samples with deuterium (a hydrogen isotope) gas under high pressure at various temperatures. Employing various temperatures aims at exploring the trap barriers and modulating the trap sites of deuterium. This facilitates understanding the deuterium mobility associated with the trap sites and how deuterium atoms convert from reversible to irreversible trap state. Herein, deuterium is utilized instead of hydrogen to augment the detection sensitivity via the TDS technique. Deuterium is considered to exhibit similar trapping and diffusional behaviors to those of hydrogen. The study results show that up to 70% of deuterium can be removed from the 22 °C-charged sample via the EO technique, which indicates that the content of reversible deuterium is several times more than that of irreversible deuterium. As the charging temperature increases, deuterium tends to diffuse into dislocations and/or micro-voids, where it becomes irreversibly trapped. Accordingly, an illustrative energy level diagram was devised to elucidate the trapping and diffusion behaviors of deuterium. This study endeavors to characterize by experimental approach the deuterium mobility associated with trap sites. Our findings contribute to a deeper understanding of hydrogen trapping in metals, advancing the design of hydrogen-resistant materials.

## 2. Experimental Procedures

A pure Fe sample was prepared from a high-purity iron (Beijing Zhongjin Yan New Material Technology Co., Ltd., Beijing, China) ingot produced by vacuum induction melting. The as-cast ingot was not subjected to any subsequent thermo-mechanical processing (e.g., rolling or forging) or post-solidification heat treatment. Samples with a diameter of 12.0 mm and a thickness of 1.0 mm were cut from this ingot and were first mechanically polished. To charge deuterium into the samples, they were immersed in a deuterium atmosphere at a pressure of 5 × 10^5^ Pa for 24 h at different temperatures. The sample was named Fe-D or Fe-D-T °C when the temperature (T) was specified. To measure the deuterium content in the sample, a TDS experiment was carried out. The sample was heated with a linear temperature ramp. For the majority of the experiments reported, a constant rate of 10 K/min was used from room temperature to 880 °C. To perform a kinetic analysis (e.g., using the Kissinger model to estimate de-trapping energy), additional experiments were conducted with ramp rates of 2, 5, 10, and 20 K/min. The deuterium released from the sample was quantified by quadrupole mass spectroscopy by recording mass 4 for the D_2_ signal (Sichuan Runtai Special Gas Co., Ltd., Deyang, China), which had been quantitatively calibrated using a standard leak system. The sample crystal structure influenced by the charged temperature was investigated by both X-ray diffraction (XRD) (Liaoning Dandong Fangyuan Instrument Co., Ltd., Dandong, China) technique and high-resolution transmission electron microscopy (HRTEM). The oxidation behaviors during the EO experiment were analyzed by X-ray photoelectron spectroscopy (XPS) (Thermo Fisher Scientific Inc., Waltham, MA, USA).

The EO experiments were carried out in a 0.1 mol/L NaOH solution (Sigma-Aldrich, St. Louis, MO, USA) by using a CHI 760E electrochemical workstation with a three-electrode cell. The Fe-D sample served as the working electrode, the Pt sheet as the counter electrode, and the Hg/HgO plate as the reference electrode. The Fe-D sample was set at a specific positive voltage, which enables the deuterium near the interface between the Fe and the NaOH solution to be continuously oxidized into deuterium ions (D^+^) in the electrolyte and removed from the sample. This creates a large concentration gradient of deuterium between the inner volume and the surface area of the sample, accelerating the diffusion of deuterium in the sample towards the surface and the continuous removal of deuterium [27]. The electrode potential should be selected within a range that is high enough to oxidize deuterium to solvated D^+^ without inducing severe corrosion of the metal. This range was determined via a cyclic voltammetry test in the same NaOH solution. The duration of the deuterium removal process is typically 4 h and can be varied as a study parameter.

## 3. Results and Discussion

**Figure 1:**
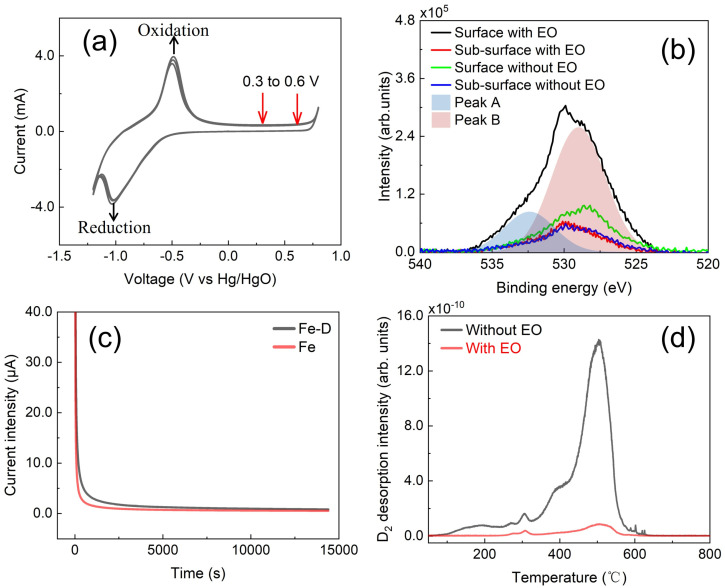
(**a**) A cyclic voltammogram for Fe-D sample in NaOH solution, (**b**) the XPS spectra of oxygen element on the surface and the sub-surface accessible by argon ion sputtering of the Fe-D samples with and without EO treatment, the peak A and B were obtained by the fitting of “surface with EO” denoted as the XPS spectrum for EO-treated Fe-D sample surface, (**c**) the I-t curves with 0.6 V for the oxidation of Fe-D and Fe samples, (**d**) the TDS spectra with 10 K/min heating rate for Fe-D samples with and without EO treatment.

To explore the reaction performance of Fe-D samples in NaOH solution, cyclic voltammetry tests (Figure 1a) were conducted within a potential window spanning from −1.2 V to 0.8 V (versus Hg/HgO electrode). In the potential range of −1.2 V to −0.1 V, two peaks associated with hydrogen reduction and OH^−^ oxidation could be discerned at approximately −0.75 V to −0.25 V. In the interval of −0.1 V to 0.6 V, a plateau region was observed, wherein Fe did not display obvious oxidation in contrast to the scenario when the potential exceeded 0.7 V. Subsequently, the EO performance of deuterium for Fe-D samples was evaluated using potentials within the plateau region, specifically 0.6 V.

Moreover, XPS measurement was conducted to verify the scarce oxidation of Fe (Figure 1b). The concentration of the oxygen (O) element within the sample depth serves as an indicator of the oxidation behavior. On the surface of the Fe-D sample with EO treatment, a distinct XPS peak in the range of 538–524 eV was ascribed to the O element. This peak was deconvoluted into Peak A and Peak B, which are located at 532.5 eV and 529.0 eV, respectively. Peak A at 532.5 eV is attributed to the O-H bond, while Peak B at 529.0 eV is assigned to the Fe-O bond. The former results from the adsorption of H_2_O or hydroxide on the surface, and the latter originates from the oxidation of Fe. In contrast, for the surface of the Fe-D sample without EO treatment, the peak intensity is considerably lower than that of the sample with EO treatment. This is attributed to both less hydroxide/H_2_O adsorption and less Fe oxidation. When the sample was etched to a sub-surface depth of approximately 15 nm, the peak intensity decreased substantially, indicating a much lower O content in the sub-surface. The two Fe-D samples, with and without EO treatment, exhibit the same peak intensity at the sub-surface. This implies that the EO treatment has no impact on the O content at a depth greater than 15 nm in the sample. Thus, it indicates that the occurrence of Fe oxidation could be neglected during the EO treatment process due to removal of deuterium in the sample. It is important to note that while the contribution of Fe oxidation to the deuterium removal process can be considered negligible, this does not exclude the occurrence of more complex surface oxide/hydroxide evolution during the EO process.

When the Fe-D sample was employed as the working electrode at a potential of 0.6 V, the current was monitored and recorded as an I-t curve (Figure 1c). It was observed that an initially relatively high current was present, which rapidly declined to a normal level within 10 s. Beyond 10 s, the current gradually diminished and asymptotically approached a stable value over a time span exceeding 3 h. A comparative study with the Fe sample was executed. The Fe sample also exhibits rapid current-drop behavior. The region with a high current within the initial 10 s might potentially be attributed to either the recording oscillation of the experimental apparatus or the surface structure of the sample. After 10 s, the Fe-D sample exhibited a higher current in the initial phase compared to the Fe sample. This may be ascribed to the oxidation of deuterium, which enhanced the current. As the reaction time elapsed, the current curve of the Fe-D sample tended to converge with that of the Fe sample. Subsequently, after 3 h, the current curves of the two samples coincided. These findings likely imply that the deuterium inside the Fe-D sample underwent oxidation, and the EO process endured for more than 3 h.

To study the effect of the EO process on the deuterium content, two identical Fe-D samples with 0.5 h air exposure of thermo-charging were chosen. One of the samples was directly subjected to deuterium measurement using TDS techniques, whereas the other sample was first processed via the EO process and then underwent deuterium measurement. The measurement results of both samples are presented in Figure 1d. The figure distinctly demonstrates that the sample which had undergone EO treatment exhibited a substantially lower peak intensity in comparison to the sample without EO treatment. The peak intensity is directly proportional to the deuterium content inside the sample. The decreased intensity implies a reduced amount of deuterium in the EO-treated sample. The reduction in quantity is as high as 85%, which is associated with the content of irreversible deuterium. One may argue that the reduction in deuterium results from a spontaneous release rather than the EO effect. To dispel this concern, we present subsequent evidence indicating the existence of non-spontaneously releasable deuterium residue within the sample. Subsequently, such non-spontaneously releasable deuterium can be removed out through the EO technique. This would provide evidence that reversible deuterium is present in the non-spontaneously releasable deuterium fraction. Furthermore, an investigation into the specific types of trap sites where the non-spontaneously releasable deuterium resides and can be removed by the EO technique would be of great interest.

**Figure 2:**
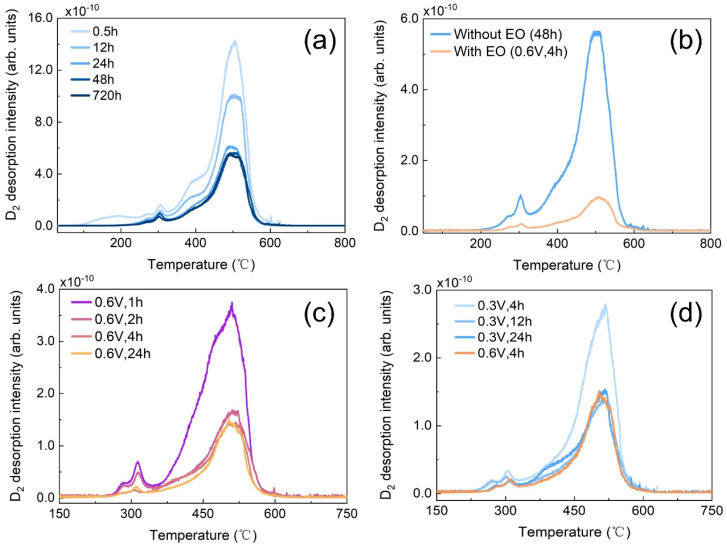
(**a**) The TDS spectra for the non-EO treatment Fe-D samples with various air exposure time, (**b**) the TDS spectra for the 48 h air-exposure Fe-D samples with and without EO treatment, (**c**) the TDS spectra for the 48 h air-exposure Fe-D samples with EO treatment by different durations, (**d**) the TDS spectra for the 48 h air-exposure Fe-D samples with EO treatment by different durations and voltages. All the TDS spectra have an identical heating rate of 10 K/min.

In order to verify whether the non-spontaneously releasable deuterium can be removed by EO technique, the work first focuses on the influence of air exposure duration on the deuterium content in the Fe-D sample without EO treatment. The TDS measurement results for the Fe-D samples with diverse air exposure times are presented in Figure 2a. In the case of the sample with an exposure time of 0.5 h, a distinct signal of deuterium released from the sample was detected when the temperature reached 100 °C. A faint peak was observed at approximately 300 °C. As the temperature exceeded 400 °C, the peak intensity escalated rapidly and exhibited a strong peak at around 500 °C. The deuterium was completely depleted when the temperature surpassed 700 °C. In the case of the sample with an exposure time of 12 h, no deuterium signal was detected within the temperature range of 100–200 °C. This confirms that the deuterium was spontaneously released into the air during the 12 h air exposure process. Both the peaks at approximately 300 °C and 500 °C still emerged, yet each demonstrated a lower intensity in comparison to that of the 0.5 h exposure sample. Furthermore, the peak intensity continued to decline with the increase in exposure time and approached a steady state for exposure times exceeding 48 h. This indicates that the deuterium release behavior persisted for approximately 48 h for the Fe-D sample after thermo-charging. The deuterium remaining in the Fe-D sample was regarded as non-spontaneously releasable deuterium. It should be noted that even if deuterium cannot be spontaneously released, when it can move freely within the sample, such deuterium can still induce embrittlement. One objective of this research is to ascertain whether the non-spontaneous releasable deuterium can be removed by the EO technique. Two identical Fe-D samples, which had been exposed to air for 48 h until reaching a steady state, were selected. One of the samples was directly measured for deuterium content using TDS techniques. In contrast, the other sample was initially processed by the EO treatment and then subjected to deuterium measurement. The measurement results of both samples are illustrated in Figure 2b. The figure clearly shows that the sample that had undergone the EO treatment exhibited a significantly lower peak intensity compared to the sample without EO treatment. This result unequivocally demonstrates that the majority of the non-spontaneously releasable deuterium can be effectively removed by the EO technique. Moreover, calculated by the peak area, the removal rate was determined to reach 70%. This indicates that the proportion of reversible deuterium in the non-spontaneously releasable deuterium fraction is as high as 70%. It should be noted that this rate is lower than the 85% obtained from the samples with an exposure time of 0.5 h. The higher removal rate in the case of shorter exposure time is attributed to the removal of spontaneously releasable deuterium. The result suggests the EO technique possesses two functions. The first one is to accelerate the removal of the spontaneously releasable deuterium. The other is to effectively remove most of the non-spontaneously releasable deuterium. This enables the calculation of the proportion of reversible deuterium within the non-spontaneously releasable deuterium fraction.

An investigation was conducted on both the oxidation duration and voltage within the EO process to optimize the EO technique. With the oxidation voltage fixed at 0.6 V, the impact of oxidation duration on the TDS spectra is illustrated in Figure 2c. It is evident that the spectrum intensity for an oxidation duration of 2 h is substantially reduced in comparison to that for 1 h. The spectrum intensity exhibits a marginal decrease for 4 h when contrasted with that for 2 h. Moreover, the spectrum intensity remains unchanged even when the duration extends to 24 h. This implies that, when the oxidation voltage is set at 0.6 V, an EO treatment with an oxidation duration of 4 h is sufficient to minimize the spectrum intensity. In other terms, the optimized oxidation duration for deuterium removal is 4 h. This outcome is in close alignment with the I-t curve presented in Figure 1b, which also demonstrates that no further deuterium can be oxidized beyond 4 h. Moreover, in the scenario where the oxidation voltage is set at the relatively low level of 0.3 V, the minimization of deuterium is attained when the oxidation duration reaches 12 h (Figure 2d). Furthermore, the influence of oxidation voltage on the TDS spectra is also depicted in Figure 2d. It is shown that, when the duration is set at 4 h, the spectrum intensity for an oxidation voltage of 0.6 V is approximately halved in comparison to that for 0.3 V. This result validates that a higher oxidation voltage accelerates the deuterium oxidation process and requires less time to reduce the deuterium residual in the sample to the minimum. Notably, the minimum deuterium levels achieved under the conditions of 0.3 V and 0.6 V are comparable. This provides evidence that the oxidation voltage does not exert a pronounced impact on the amount of deuterium removed. The deuterium removal process is regarded as consisting of two steps, namely diffusion within the sample bulk and oxidation on the sample surface [28]. By augmenting the oxidation rate on the sample surface through an increase in oxidation voltage, the rate of deuterium removal can be significantly enhanced. These results indicate that for the EO process on Fe samples, the surface electro-oxidation reaction may be the rate-determining step under the studied conditions.

**Figure 3:**
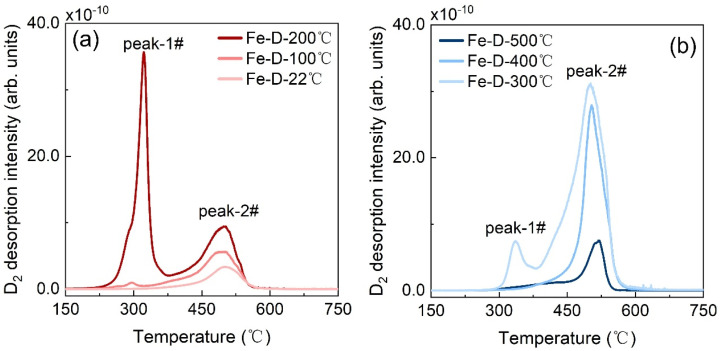
The TDS spectra for the non-EO treatment and 48 h air-exposure Fe-D samples with charging at different temperatures (**a**) from 22 to 200 °C and (**b**) 300 to 500 °C. All the TDS spectra have an identical heating rate of 10 K/min.

Aiming to further probe the evolution of reversible deuterium content, we endeavored to engineer the deuterium residing in diverse trap sites through thermo-charging at varying temperatures. To this end, the effect of charging temperature on the TDS spectra (Figure 3a,b) was examined for Fe-D-T °C samples in a steady state. In the case of thermo-charging at 22 °C corresponding to the Fe-D-22 °C sample, only a single peak emerged at approximately 500 °C in the TDS spectrum. This peak is designated as peak-2#. When thermo-charging was conducted at 100 °C, the intensity of peak-2# increased in contrast to that of the Fe-D-22 °C sample. Moreover, the Fe-D-100 °C sample has a new yet weak peak located at around 300 °C. This peak is named as peak-1#. As the thermo-charging temperature was further elevated from 100 °C to 500 °C, both peak-1# and peak-2# initially increased and then decreased. Peak-1# exhibited its maximum intensity for the Fe-D-200 °C sample but vanished when the charging temperature exceeded 400 °C. Peak-2# attained its maximum intensity for the Fe-D-300 °C sample.

**Figure 4:**
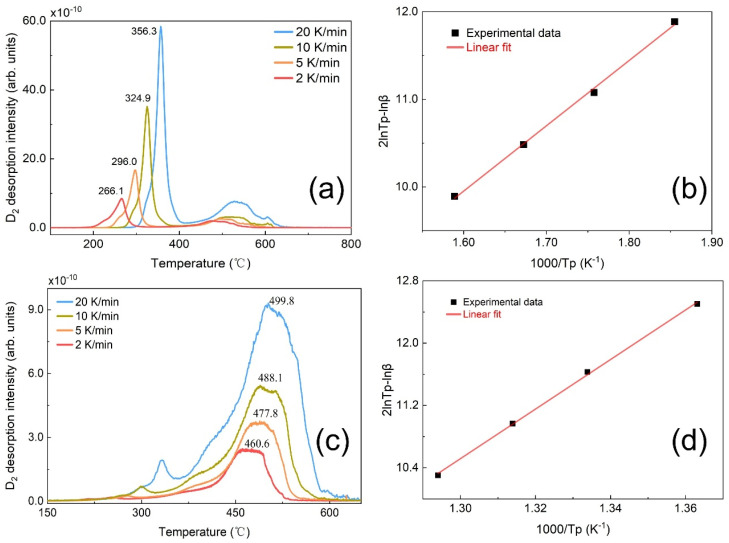
For the non-EO treatment and 48 h air-exposure Fe-D-200 °C samples (**a**,**b**) and Fe-D-300 °C samples (**c**,**d**), the TDS spectra with the indicative heating rates (**a**,**c**) and the data points as well as their linear fit for $2\ln T_{p}-\ln\beta$ versus $\frac{1000}{T_{p}}$ (**b**,**d**), where the T_p_ denotes the peak temperature corresponding to heating rate β.

In the subsequent two sections, we attempted to first identify the origins of the peaks in TDS spectra and then elucidate the rationale behind the evolution of peak intensities. Regarding the former, it is assumed that peak-1# and peak-2# in the TDS spectra originate from the release of deuterium from two specific traps. To characterize the nature of these traps, we employed the conventional Kissinger model to estimate the de-trapping energy. The Kissinger model is expressed by the formula [29]:

$$\ln(\frac{\beta }{T_{p}^{2}})=\ln(A\frac{R}{E_{dt}})-\frac{E_{dt}}{R}\frac{1}{T_{p}}$$

where β represents the heating rate (in K/min) and T_p_ denotes the temperature (in K) corresponding to the TDS peak of interest. A is a constant that depends on material parameters and trap site characteristics. R is the universal gas constant, and E_dt_ is the de-trapping energy of gas within the metal. We anticipated that by varying the heating rate, the de-trapping energy could be calculated from the slope of a 2lnT_p_ − lnβ versus 1000/T_p_ plot. For peak-1#, the de-trapping energy was calculated on the basis of representative sample Fe-D-200 °C, which exhibited a strong intensity. As depicted in Figure 4a, when the heating rate was increased from 2 K/min to 20 K/min, the temperature of the peak-1# shifted significantly from 266.1 °C to 356.3 °C. The plot of 2lnT_p_ − lnβ versus 1000/T_p_ is presented in Figure 4b, enabling the extraction of the de-trapping energy from its slope [30]. The calculated de-trapping energy for peak-1# was 0.65 eV. Similarly, from Figure 4c, the de-trapping energy for peak-2# was determined to be 2.76 eV. These energies are considerably higher than the reported values [31]. This discrepancy can be attributed to the limitations of the Kissinger model. This model assumes that gas desorbs directly from the residing trap, neglecting any diffusion within the sample. In fact, the thermal desorption process likely involves an initial diffusion of gas from one trap to another within the sample, followed by desorption from the sample surface. This diffusion retards the deuterium desorption from the sample, resulting in desorption occurring at a higher temperature. Consequently, the calculated de-trapping energy is higher than expected. Despite the calculated values not being entirely accurate, they provide insights into the two types of traps with different energies present in the sample. Peak-1# corresponds to a shallow trap, while peak-2# is associated with a deep trap. On the basis of analysis on the microstructure of Fe metal and the relevant literature, we hypothesize that the shallow trap could be associated with dislocations, and the deep trap may be related to micro-voids [24,32,33]. The interstitial sites are not linked to either peak-1# or peak-2# due to their shallower traps [34]. Even if deuterium is initially located in the interstitial sites, it would diffuse into the dislocations or micro-voids during the heating process in the TDS experiment. This diffusion results in the deuterium exhibiting a de-trapping energy corresponding to that of the dislocations or micro-voids.

**Figure 5:**
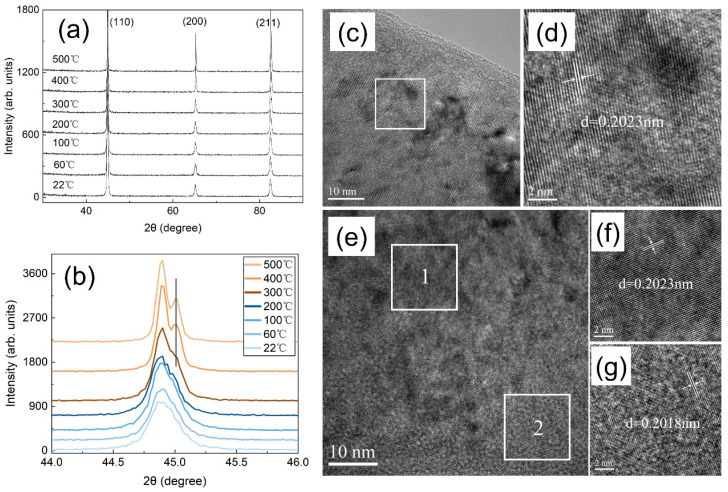
The XRD spectra (**a**) in a wide range of 10–90 degree and (**b**) in a narrow range of 44.0–46.0 degree for the Fe-D samples with charging at different temperatures, HRTEM images for Fe-D-22 °C (**c**,**d**) and Fe-D-500 °C (**e**–**g**).

As for the latter, the reason for the evolution of peak intensities is as follows. For peak-1#, no signal was detected in the Fe-D-22 °C sample, which can be attributed to the barrier impeding the diffusion of deuterium into the dislocations. With the increase in charging temperature to 100 °C and 200 °C, the deuterium possessed more energy to overcome the barrier and occupy the dislocations. A higher charging temperature enabled more deuterium to reside in the dislocations, resulting in a higher peak intensity for the Fe-D-200 °C sample compared to that of Fe-D-100 °C. When the charging temperature reached 300 °C, the peak intensity decreased. This can be explained, on one hand, by the instability of high-energy deuterium in the dislocations. The deuterium escaped from the dislocations with less energy trapped, while some deuterium remained in the dislocations with deeper energy trapped, which was associated with the shift of the peak-1# position to a higher temperature. When the charging temperature was further increased to 400 °C and 500 °C, the deuterium had excessive energy to be retained in the dislocations. On the other hand, another possible reason may be related to the decrease of dislocations caused by the structural reorganization due to atomic diffusion during the thermos-charging process. This is corroborated by both XRD and HRTEM results (Figure 5). The XRD data reveals that a single broad peak bifurcates into two peaks, a phenomenon that becomes pronounced when the charging temperature exceeds 300 °C. Meanwhile, the HRTEM results demonstrate that the Fe-D-500 °C sample exhibits two distinct lattice distances, whereas it was not easy to notice these in the Fe-D-22 °C sample. These findings imply that the variation of the TDS spectrum with respect to the charging temperature can be rationalized by the significant microstructural alterations that occur when the charging temperature surpasses 300 °C. For peak-2#, the evolution of its intensity can be explained in a similar manner to that of peak-1#. The following comparison should be noted: peak-2# intensities for both Fe-D-22 °C and Fe-D-100 °C samples were higher than that of peak-1#. This can be accounted for by two factors. Firstly, the diffusion of deuterium into the micro-voids has a lower energy barrier than that into the dislocations. Secondly, more trap sites are located near the micro-voids since the micro-voids are arranged in a disorderly manner. These trap sites possess a lower barrier, permitting more deuterium to be retained. During the heating process in the TDS measurement, the retained deuterium would first diffuse into the adjacent micro-voids and then desorb out of the sample.

**Figure 6:**
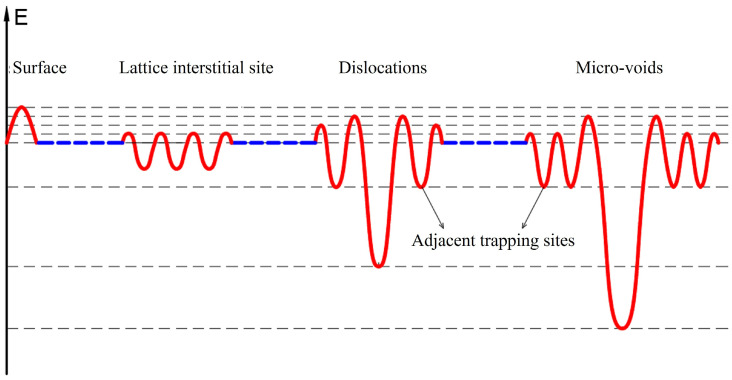
The energy-level diagram of deuterium in Fe system corresponding to various trap sites, such as lattice interstitial site, dislocations, micro-voids, and the adjacent trap sites of dislocations or micro-voids.

Accordingly, the energy levels of deuterium in the Fe system corresponding to various trap sites, such as lattice interstitial site, dislocations, micro-voids, and the adjacent trap sites of dislocations or micro-voids were concisely illustrated in Figure 6. The surface exhibits a significant energy barrier as one factor to impede the spontaneous release of deuterium at room temperature. It is postulated that the non-spontaneously releasable deuterium is located at the dislocations, micro-voids, and the adjacent trap sites of dislocations or micro-voids [35]. The dislocations are associated with a relatively deep trap, and the micro-voids with an even deeper trap, whereas the adjacent sites correspond to shallower traps. The region proximal to the micro-voids is characterized by a higher degree of disorder compared to that near the dislocations. Hence, the shallow trap quantity in the vicinity of the micro-voids is greater than that around the dislocations.

**Figure 7:**
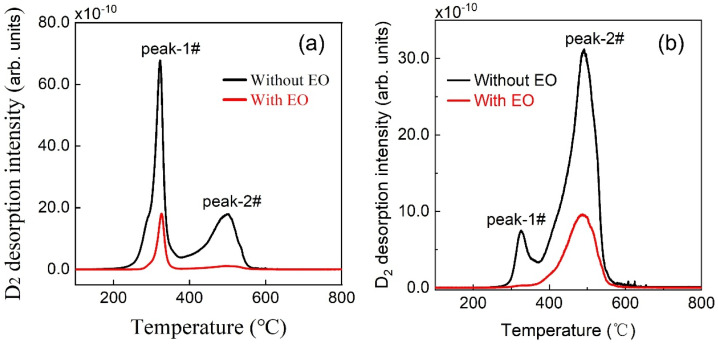
TDS spectra with 10 K/min heating rate for the 48 h air-exposure (**a**) Fe-D-200 °C and (**b**) Fe-D-300 °C samples with and without the EO treatment.

On the basis of the above energy level diagram, the influence of EO treatment on the residual deuterium within the Fe-D sample was explained. Two representative samples, namely Fe-D-200 °C and Fe-D-300 °C, were chosen, as the former exhibits the maximum intensity of peak-1# while the latter shows the maximum intensity of peak-2#. In the case of the Fe-D-200 °C sample (as depicted in Figure 7a), both peak-1# and peak-2# remarkably decline subsequent to the EO treatment. A substantial amount of deuterium is still desorbed from the dislocations, yet a lesser quantity is desorbed from the micro-voids. As illustrated in the diagram of energy level, the micro-voids possess a deeper trap compared to the dislocations. If deuterium were present both in the dislocations and the micro-voids, the removal of deuterium from the dislocations should be completed first. However, this situation does not conform to the experimental results. A plausible explanation pertains to the fact that the deuterium is situated in the adjacent trap sites rather than the micro-voids. Such deuterium was extracted out of the sample during the EO treatment, and consequently, less deuterium diffuses into the micro-voids, resulting in a relatively low intensity for peak-2#. Regarding peak-1#, some deuterium remains in the adjacent trap sites, potentially due to a relatively high energy barrier for deuterium removal and/or the presence of deep-trap dislocations that hinders deuterium removal. This leads to a higher intensity of peak-1# compared to that of peak-2#. For the Fe-D-300 °C sample with EO treatment (as shown in Figure 7b), only a negligible amount of deuterium is desorbed from the dislocations, while a significant amount is desorbed from the micro-voids. Peak-1# nearly vanishes after the EO treatment. This can be accounted for by the fact (Figure 5) that 300 °C-charging induces microstructural alterations, such as the elimination of most of the defects related to dislocations. Additionally, peak-2# still displays a considerable intensity even for the sample treated by the EO technique. This might be attributed to the high energy of the deuterium, which is a consequence of charging at the elevated temperature of 300 °C, enabling the deuterium to overcome the energy barrier and diffuse into the micro-voids. Deuterium trapped in micro-voids leads to an irreversible state. In consequence, in the case of low-temperature charging, deuterium mostly resides in trap sites adjacent to dislocations or micro-voids and exists as a reversible state. In contrast, as part of these findings, deuterium receives sufficient energy from high-temperature charging to overcome the energy barrier, enabling it to diffuse into dislocations or micro-voids and thus transforms to an irreversible state.

## 4. Conclusions

An integrated approach combining EO and TDS was employed to characterize both reversible and irreversible deuterium in Fe samples. The trap sites of deuterium were modulated by varying the deuterium charging temperature. The EO technique not only accelerates the expulsion of spontaneously releasable deuterium but also effectively removes most of the non-spontaneously releasable deuterium. The study results provide evidence that the proportion of reversible deuterium in the non-spontaneously releasable deuterium fraction is as high as 70%. Under low-temperature charging conditions, deuterium predominantly localizes in trap sites adjacent to dislocations or micro-voids, existing as a reversible state. Conversely, high-temperature charging provides deuterium with sufficient energy to surmount the energy barrier. This allows it to diffuse into dislocations or micro-voids, thereby transitioning into an irreversible state. This work enriches our understanding of the interactions between hydrogen and Fe materials, thereby reinforcing the fundamental theories underlying hydrogen embrittlement.

## Data Availability

The original contributions presented in this study are included in the article. Further inquiries can be directed to the corresponding author.
